# Authors’ Reply: Critical Limitations in Comparing ChatGPT and DeepSeek for Orthopedic Assessment

**DOI:** 10.2196/91470

**Published:** 2026-03-17

**Authors:** Chirathit Anusitviwat, Sitthiphong Suwannaphisit, Jongdee Bvonpanttarananon, Boonsin Tangtrakulwanich

**Affiliations:** 1 Prince of Songkla University, 15 Karnjanavanich Road, Hat Yai, Songkhla, 90110, Thailand, 66 74451601; 2 Navamindradhiraj University, Bangkok, Bangkok, Thailand

**Keywords:** ChatGPT, large language model, LLM, orthopedic, multiple-choice question, MCQ

We thank you for the useful and constructive comments [1] on our article “Comparing ChatGPT and DeepSeek for Assessment of Multiple-Choice Questions in Orthopedic Medical Education: Cross-Sectional Study” [2]. This reply aims to address the concerning points that were brought up in the letter to the editor.

## Misinterpretation of Reliability Statistics

According to our study, we administered the multiple-choice questions (MCQs) for ChatGPT and DeepSeek on a separate day. All data from the two large language models (LLMs) were measured by two assessors. Although two assessors were used for each LLM, the reported Cohen κ coefficient values represent within-model interrater reliability, not interrater reliability between the two LLMs [3]. Therefore, describing these results as agreement between the two models is inaccurate.

## Linguistic Ambiguity and Generalizability

All MCQs used in our study were administered in English. No Thai language inputs or translations were used. Therefore, the performance differences between the two models reflect the model performance on English language medical questions rather than variability due to language translation or non-English linguistic processing.

## Reproducibility and Interface Transparency

All models in our study were accessed via web-based user interfaces (UIs), not application programming interfaces. We acknowledge that web-based UIs may be subject to updates and lack version control. However, the web-based version of ChatGPT is easy to access and requires no software installation. It also allows quick testing and exploration without technical or cost barriers, making it well-suited for nontechnical users and educational studies [4]. Therefore, we used the web-based UI in our study.

## Risk of Data Contamination

Even though these MCQs have been used for more than 5 years, the MCQs used in our study are from private orthopedic examinations. Thus, we believe that these items would not appear in public sources. Future research using newly created MCQs may be better for assessing the capability or efficacy of LLMs.

## Data Reporting Discrepancy

Upon re-examination, we confirm that the correct accuracy for the pelvic and spine injury category (n=19) using the Reason function is indeed 16 of 19, corresponding to approximately 84.2%. The value of 68.8% reported in Table 2 was a typographical error. This error has been corrected through a published corrigendum [5].

## Abbreviations

LLM: large language model
MCQ: multiple-choice question
UI: user interface

## References

[1] Ayas O, Acar A (2026). Critical limitations in comparing ChatGPT and DeepSeek for orthopedic assessment. JMIR Form Res.

[2] Anusitviwat C, Suwannaphisit S, Bvonpanttarananon J, Tangtrakulwanich B (2025). Comparing ChatGPT and DeepSeek for assessment of multiple-choice questions in orthopedic medical education: cross-sectional study. JMIR Form Res.

[3] McHugh ML (2012). Interrater reliability: the kappa statistic. Biochem Med (Zagreb).

[4] Park CR, Heo H, Suh CH, Shim WH (2025). Uncover this tech term: application programming interface for large language models. Korean J Radiol.

[5] Anusitviwat C, Suwannaphisit S, Bvonpanttarananon J, Tangtrakulwanich B (2026). Correction: comparing ChatGPT and DeepSeek for assessment of multiple-choice questions in orthopedic medical education: cross-sectional study. JMIR Form Res.

